# Review of Recent Developments in the Fabrication of ZnO/CdS Heterostructure Photocatalysts for Degradation of Organic Pollutants and Hydrogen Production

**DOI:** 10.3390/molecules28114277

**Published:** 2023-05-23

**Authors:** Santhosh Kumar Nadikatla, Vinod Babu Chintada, Thirumala Rao Gurugubelli, Ravindranadh Koutavarapu

**Affiliations:** 1 Chemistry Division, Department of Basic Sciences and Humanities, GMR Institute of Technology, Rajam 532127, Andhra Pradesh, India; nadikatla.56@gmail.com; 2 Department of Mechanical Engineering, GMR Institute of Technology, Rajam 532127, Andhra Pradesh, India; vinodbabu.chintada@gmail.com; 3 Physics Division, Department of Basic Sciences and Humanities, GMR Institute of Technology, Rajam 532127, Andhra Pradesh, India; 4 Department of Robotics Engineering, College of Mechanical and IT Engineering, Yeungnam University, Gyeongsan 38541, Republic of Korea

**Keywords:** ZnO/CdS heterostructures, photocatalysis, synthesis methods, hydrogen production

## Abstract

Researchers have recently paid a lot of attention to semiconductor photocatalysts, especially ZnO-based heterostructures. Due to its availability, robustness, and biocompatibility, ZnO is a widely researched material in the fields of photocatalysis and energy storage. It is also environmentally beneficial. However, the wide bandgap energy and quick recombination of the photoinduced electron–hole pairs of ZnO limit its practical utility. To address these issues, many techniques have been used, such as the doping of metal ions and the creation of binary or ternary composites. Recent studies showed that ZnO/CdS heterostructures outperformed bare ZnO and CdS nanostructures in terms of photocatalytic performance when exposed to visible light. This review largely concentrated on the ZnO/CdS heterostructure production process and its possible applications including the degradation of organic pollutants and hydrogen evaluation. The importance of synthesis techniques such as bandgap engineering and controlled morphology was highlighted. In addition, the prospective uses of ZnO/CdS heterostructures in the realm of photocatalysis and the conceivable photodegradation mechanism were examined. Lastly, ZnO/CdS heterostructures’ challenges and prospects for the future have been discussed.

## 1. Introduction

Globally, increased urbanization and industrialization have elevated ecological issues. These problems lead to air and water effluence, putting world health in danger [1,2,3,4,5]. To generate completed goods, numerous companies, such as chemical factories, refineries, and plastics manufacturers, use hazardous and organic pollutants and then discharge them directly into natural waterways and rivers [6,7,8]. These contaminants can stop bacteria, insects, and microscopic plants in the water sources from degrading as they would typically do. Poisonous and extremely rigorous contaminants such as azo dye, aromatic chemicals, medicines, and others are changing the biological oxygen demand in water, which harms aquatic life. All these hazardous pollutants also cause allergic dermatitis, skin irritation, and some of them can cause cancer [9,10,11,12]. As a result, these harmful contaminants have an adverse effect on human health in addition to harming aquatic life. Therefore, treating the polluted wastewater is a crucial environmental concern nowadays [13,14,15]. Various chemical, physical, and biological procedures are employed to remove hazardous contaminants from wastewater. These include chemical oxidation, photocatalytic treatment, anaerobic, activated muds, flocculation, coagulation, adsorption, reverse osmosis, sedimentation, ultrafiltration, and so on [16]. Out of all of them, photocatalytic treatment is a technique which completely removes the toxic pollutants from wastewater with a high degradation efficiency at a low cost and minimum processing time, making it a promising method [17,18,19,20]. 

**Figure 1:**
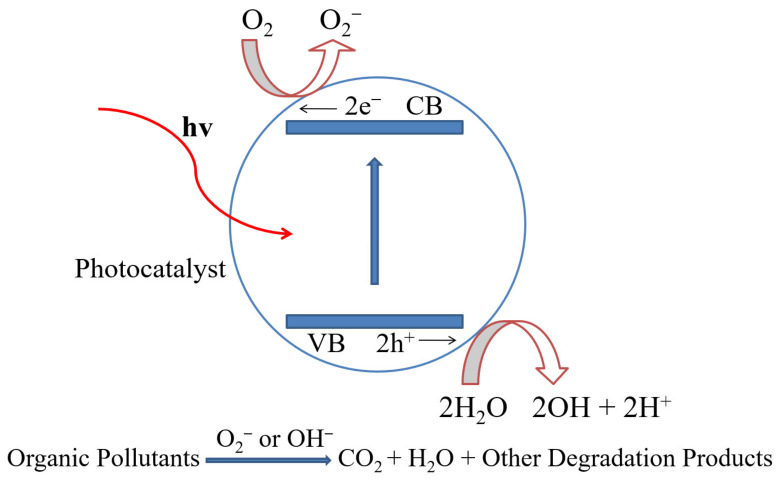
Schematic representation of photocatalysis process.

In the photocatalysis process, transition metal oxides and semiconductors are commonly used as heterogeneous photocatalysts. They have a prohibited energy gap that requires visible light to migrate electrons from the filled VB to the CB. This excites a positive hole in the VB and a free electron in the CB, resulting in an electron–hole pair [21,22,23]. The ultimate purpose of the photocatalyst design is to allow reactions between the produced holes and reductant, leading to the production of an oxidized product, while simultaneously facilitating the interaction of excited electrons to produce a reduced product. A schematic depiction of the photocatalysis process is depicted in Figure 1. The surface of the semiconductor undergoes an oxidation reduction reaction as a result of the formation of holes and electrons. The positive holes combine with the moisture on the surface to form hydroxyl radicals during the oxidation reaction. A detailed mechanism of the photocatalysis process is given below:

Photocatalysts+hv→e+h

$$h^{+}+H_{2}O\to H^{+}+{\mathrm{OH}}^{+}$$

$$h^{+}+{\mathrm{OH}}^{-}\to {\mathrm{OH}}^{+}$$

$$e^{-}+O_{2}\to O_{2}^{-}$$

$$2e^{-}+O_{2}+2H^{+}\to H_{2}O_{2}$$

$$e^{-}+H_{2}O_{2}\to {\mathrm{OH}}^{+}+{\mathrm{OH}}^{-}$$

$$\mathrm{Organic}+\mathrm{OH}\to {\mathrm{CO}}_{2}+H_{2}O+\mathrm{Other}\;\mathrm{degradation}\;\mathrm{products}$$

In the photocatalysis process, semiconductor materials have been extensively considered for the degradation of pollutants from wastewater, and the splitting of hydrogen and oxygen from water for clean fuel. TiO_2_, ZnO, SnO_2_, BiVO_4_, InVO_4_, WO_3_, Fe_2_O_3_, CdS, ZnS, and SnS are the various semiconductor materials which can be employed as an effective catalyst in photocatalysis [24,25,26,27,28,29,30,31,32,33]. However, recent reports have revealed that the photocatalytic activity of these individual semiconductors was limited for practical applications. This is because the wide bandgap semiconductors have a limited visible-light absorption capacity and rapid recombination of charge carriers, whereas the narrow bandgap semiconductors still suffer from instability. Several methods, including the doping of metal ions and the development of binary or ternary composites, have been employed to overcome these drawbacks [34,35]. Among these techniques, constructions of heterostructures with a wide bandgap and a narrow bandgap semiconductor have been recognized as one of the most effective ways for enhanced visible-light-driven photocatalysis [36].

Recent research has revealed that ZnO/CdS heterostructures outperformed pristine ZnO and CdS nanostructures in terms of photocatalytic performance under visible-light irradiation [37,38,39]. Because of their distinctive electronic band structure, changed bandgap, and high charge transfer efficiency, ZnO-based semiconductor nanostructures have been employed as efficient photocatalytic materials for the breakdown of organic pollutants and chemical processes such as hydrogen or oxygen synthesis [40,41]. On the other hand, because of its excellent visible-light absorption capabilities, narrow bandgap (2.4 eV), and appropriately negative flat band potential, CdS is a well-known semiconductor material for photocatalytic applications [42]. Moreover, the ZnO and CdS crystal structures are well matched, allowing for the necessary reduction in the rate at which photoinduced charge carriers recombined [43,44]. The scientific frontiers of electrocatalysis and photocatalysis have recently gained a lot of attention. The electrochemical reactions in heterogeneous catalysis take place at the electrode–electrolyte interface, where the electrode serves as both a catalyst and an electron donor/acceptor [37,45]. Therefore, ZnO/CdS heterostructures have been considered as the most researched materials for the photocatalysis process owing to their enhanced optical and catalytic properties compared to the bare ZnO and CdS. Due to its nontoxicity, high stability, relative affordability, lack of mass transfer resistance, ability to operate in ambient conditions, and most importantly, the potential to decompose recalcitrant organic pollutants into less harmful compounds in a short span, photocatalysis using ZnO/CdS heterostructures has been recognized as one of the most fascinating and potentially effective methods [46,47]. In this context, the use of ZnO/CdS heterostructures in photocatalysis has drawn considerable attention from a wide range of researchers and academicians since it provides an effective and environmentally friendly solution to wastewater management [48,49].

Recently, He et al. synthesized binary CdS/ZnO heterostructures by a facile aqueous chemical process and found that the optimized CdS/ZnO heterostructures (CZ-3) exhibited a superior hydrogen production rate and dye degradation efficiency than the bare ZnO and CdS [37]. According to Lu et al., the hydrothermally grown ZnO/CdS heterostructures on FTO substrate have shown a 105 times higher photocatalytic hydrogen production rate (7669 μmol/g/h) than pure ZnO with a highest cyclic stability of 92.02% after 5 cycles for the optimized sample (ZS3) [47]. Sadhasivam et al. achieved a highest photocurrent density of 3.6 mA/cm^2^ for Cd-doped ZnO/CdS heterostructures, which is 10 times higher than pristine ZnO [49]. Guo et al. reported the high rate of hydrogen generation and stability with the ZnO/CdS core/shell heterostructures [38]. Arun et al. demonstrated that the Sn (0.6%)-doped ZnO/CdS displayed a higher photodegradation activity over RB 160 dye in 120 min and the •OH radicals are the major contributors in the photodegradation process [39]. Nandi and Das reported that the ZnO/CdS/CuS ternary heterostructure exhibited a better photodegradation efficiency than pristine ZnO and binary ZnO/CdS heterostructures [50].

ZnO/CdS heterostructures still have some opportunity to grow in terms of their photocatalytic performance, though. The effectiveness of ZnO/CdS heterostructure photocatalysts has recently been improved by a variety of methods, such as morphological control, doping, cocatalyst loading optimization, development of ternary heterostructures, etc. [51,52,53,54,55]. Thus, a review of recent research advances is required to offer a clear roadmap for forthcoming breakthroughs. ZnO/CdS heterostructure fabrication techniques and modification tactics have been thoroughly covered in this paper. In addition, the plausible mechanism and potential applications of the ZnO/CdS heterostructure photocatalyst are also summarized. In conclusion, the future of the ZnO/CdS heterostructure photocatalyst is presented. This review is anticipated to offer some helpful advice and illumination for enhancing the performance of heterojunction composites.

## 2. Development of ZnO/CdS Heterostructure Photocatalysts

Since the synthesis process might affect their potential usage in numerous applications, it is essential to understand the parameters influencing the characteristics and performance of the photocatalysts. The photocatalytic activity of the catalyst could be improved by the diminished recombination rate of photogenerated charge carriers, boosted visible-light absorption capability, and increased specific surface area of the photocatalyst. Formation of heterostructures is one of the successful ways for improved photocatalytic performance than the usage of a single semiconductor [56]. When two semiconductors with varying lattice spacing are combined, the lattice may mismatch, creating flaws that act as a hotspot for the recombination of photoinduced e^−^–h^+^ pairs, which limits the efficiency of the photocatalyst. There are many considerations to make while fabricating ZnO/CdS heterostructures. To begin, the semiconductors must have the proper band orientation [57]. Additionally, semiconductors should be included under that heading because their distinct physicochemical features are completely utilized [35]. Selecting the right fabrication strategy becomes essential if optimizing the nanocomposite becomes the primary objective. It has been discovered that the performance of the photocatalyst might be significantly influenced by the preparation method. In general, preparation techniques used for the development of diverse photocatalysts may affect their performance with a varying size, morphology, and electronic band structure. Particularly, in the case of ZnO/CdS heterostructures, numerous low-cost and environmentally friendly preparation techniques have been employed to acquire improved photocatalytic activity with high stability. In this section, we review the main fabrication methods for ZnO/CdS heterostructures, viz., coprecipitation, microwave-assisted coprecipitation, microwave irradiation, hydrothermal, solvothermal, wet chemical, sonochemical, SILAR, chemical bath deposition, and atomic layer deposition [50,58,59,60,61,62,63,64,65]. Thorough investigation is needed to understand the fundamental mechanisms that govern the features and performance of the photocatalysts and to enhance the synthesis methods for the development of ZnO/CdS heterostructures. Table 1 provides a summary of the ZnO/CdS heterostructure fabrication processes, and the details are addressed below.

### 2.1. Chemical CoPrecipitation/Microwave Assisted CoPrecipitation Method

**Figure 2:**
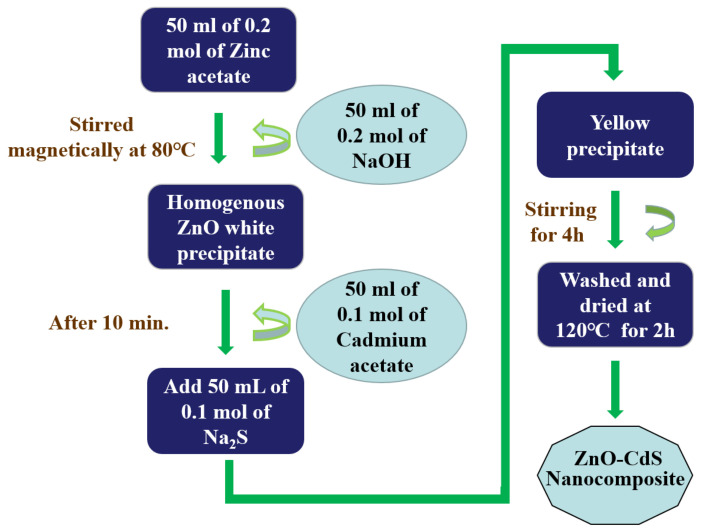
Schematic illustration of fabrication process of ZnO/CdS heterostructures by co-precipitation method.

The chemical coprecipitation method was considered as one of the most suitable techniques for the fabrication of ZnO/CdS heterostructures due to the various advantages such as easy processing, low cost, and quick reaction time. Gurugubelli et al. prepared ZnO/CdS heterostructures by the coprecipitation method for a higher photocatalytic performance under solar light [40]. Saxena et al. prepared ZnO/CdS heterostructures which significantly improved the photocatalytic activity [66]. He et al. fabricated CdS/ZnO heterostructures using the chemical precipitation method and achieved a superior photocatalytic activity over Congo red dye and excellent hydrogen production rate [57]. Rao et al. reported the synthesis of Fe-doped ZnO/CdS nanocomposites via the chemical precipitation method and observed a better photodegradation efficiency using MB dye [67]. However, the morphology of the heterostructures was well controlled but the nonuniform distribution of particles and large grain size was the major drawback of the chemical precipitation route. To overcome this drawback, the researchers adopted microwave assistance to the precipitation method, which controls the reaction time, size, and shape of the heterostructures. Chen et al. synthesized a CdS/ZnO heterostructure with a controlled morphology for improved hydrogen production. Furthermore, the H_2_ production efficiency was improved by forming a ternary GO/CdS/ZnO heterostructure [58]. Figure 2 depicts the schematic illustration of the fabrication process of ZnO/CdS heterostructures by the coprecipitation method. Finally, the coprecipitation method is a simple and scalable technique with uniform particle size distribution, which allows for the synthesis of large quantities of nanocomposites in a cost-effective manner. It also provides a high degree of control over the composition of the nanocomposites, which allows for the tuning of their properties and performance. However, there are a few limitations of the coprecipitation method which include controlling the particle size, secondary phase formation, agglomeration of nanoparticles, and sensitivity to reaction conditions. Overall, the coprecipitation method is a powerful tool for the preparation of ZnO/CdS heterostructure nanocomposites with controlled properties and performance, which has significant potential for various applications in fields such as photocatalysis, electronics, and biomedicine. While coprecipitation is a widely used method for the synthesis of nanoparticles, it is important to consider its limitations and potential drawbacks when selecting a method for a specific application.

### 2.2. Microwave Method

**Figure 3:**
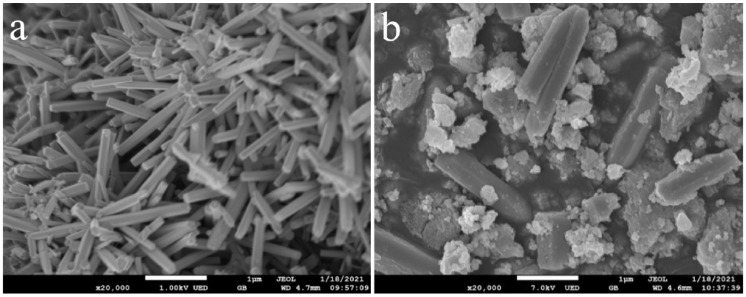
SEM micrographs of (**a**) ZnO, (**b**) ZnO/CdS synthesized by microwave method [69].

Generally, microwave synthesis is known for a fast reaction time, homogeneous particle distribution, and morphology control. Microwave synthesis has been employed to create ZnO/CdS heterostructures because it combines the advantages of speed and homogeneous heating of the precursor materials. Due to the penetrating nature of microwave irradiation, the reaction solution can be heated uniformly. A controlled size distribution of crystallites is produced as a result of homogeneous nucleation and rapid growth of the crystallites [68]. When compared to other conventional techniques, microwave synthesis has the advantage of a faster reaction time because of the combined forces created by the microwave’s electric and magnetic components, which lead to friction and molecular collisions. Revathi et al. fabricated ZnO/CdS heterostructures with an improved H_2_ production [59]. Huo and Chen synthesized CdS/ZnO heterostructures with a high hydrogen production rate [69]. Figure 3 displays the SEM micrographs of (a) ZnO and (b) ZnO/CdS nanostructures synthesized by the microwave method.

In conclusion, microwave synthesis is a promising technique to fabricate heterostructure nanocomposites, offering advantages such as faster reaction rates, energy efficiency, improved control over reaction conditions, and scalability. These benefits make microwave synthesis a preferred method for producing high-quality and functional nanocomposites. However, the demerits, such as uneven heat distribution, defect formation, and safety concerns, limit the utility of this method. Proper experimental design and control over reaction conditions can help mitigate the disadvantages of microwave synthesis.

### 2.3. Hydrothermal Method

**Figure 4:**
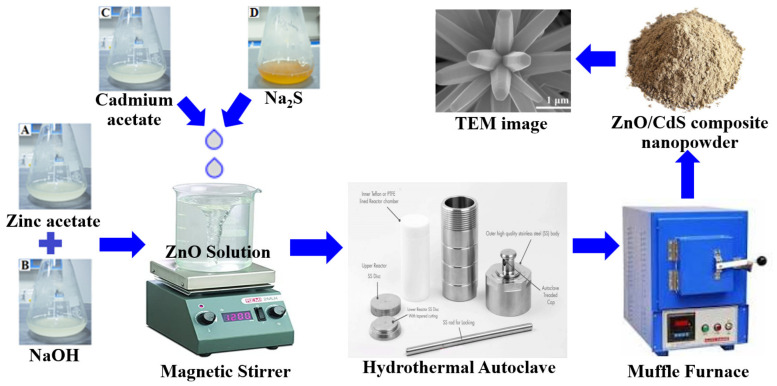
Schematic illustration of ZnO/CdS heterostructures synthesized by hydrothermal method.

The hydrothermal method is the right choice when a high degree of crystallinity, homogeneous stoichiometry, and defect-free nanostructure are required. It is frequently used to create ZnO/CdS heterostructures because of its many advantages, such as simple processing, great crystallinity, high purity, and the lack of a need for high temperature calcination [70]. The photocatalysts’ size, morphology, and bandgap can be easily modified by varying the experiment parameters such as the reaction time, temperature, pH, and other variables to obtain the desired results [71]. In turn, it leads to a more uniform crystal growth and reduced defect formation, which directly affect the efficiency of ZnO/CdS heterostructures. Recent research has demonstrated the outstanding photocatalytic performance of ZnO/CdS heterostructures in the hydrothermal process. Senasu et al. developed ZnO/CdS heterostructures for the photodegradation of antibiotic (OFL) and reactive azo dye (RR141) [60], while Guo et al. fabricated core/shell heterostructures for stable and efficient hydrogen production [38]. Zhao et al. synthesized r-GO-supported ZnO/CdS heterostructures for the efficient removal of Cr ions from an aqueous solution [72]. Adegoke et al. prepared a CdS/ZnO heterostructure photocatalyst for the decolorization of RhB under activated sunlight [73]. Lu et al. fabricated S-scheme ZnO/CdS heterostructures for improved H_2_ production [47]. It was observed that the morphology of the heterostructures was either nanoparticles (0D) or nanorods (1D). In addition, the bandgap energy was also well tuned by forming a heterojunction between the interface of ZnO and CdS. Finally, the hydrothermal method can offer the desired photocatalytic characteristics with a controlled morphology and tunable bandgap. A schematic illustration of ZnO/CdS heterostructures synthesized by the hydrothermal method is displayed in Figure 4.

Hydrothermal synthesis provides a high degree of control over the size and morphology of ZnO/CdS heterostructure nanocomposites, which is essential for achieving specific properties and applications. Particularly, the hydrothermal method offers high crystallinity, which leads to a better photocatalytic performance and stability due to the coactive effect between ZnO and CdS. Overall, the hydrothermal synthesis of ZnO/CdS heterostructure nanocomposites makes it an attractive method for various applications in fields such as photocatalysis, energy conversion, and environmental remediation. While hydrothermal synthesis has several advantages for the preparation of heterostructure nanocomposites, it also has some potential disadvantages to consider, including limited material options, a complex experimental setup, slow reaction rates, difficulty in controlling stoichiometry, and limited control over crystal orientation. However, these limitations can be addressed by careful experimental design and optimization, making hydrothermal synthesis a useful method for the preparation of advanced nanocomposites.

### 2.4. Solvothermal Method

**Figure 5:**
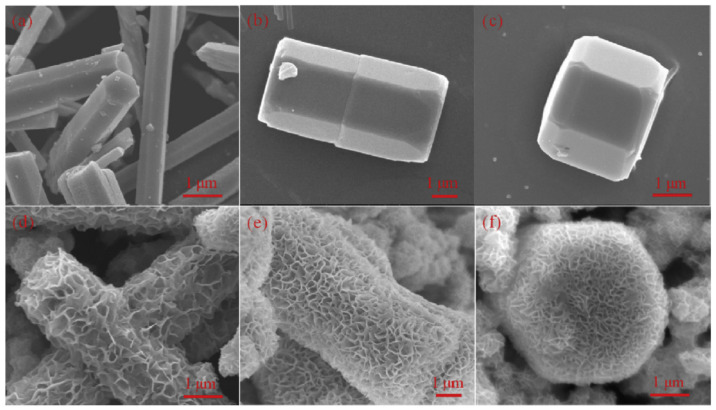
SEM micrographs of (**a**,**d**) rod-like ZnO, CdS@ZnO-3, (**b**,**e**) short rod-like ZnO, CdS@ZnO-2, and (**c**,**f**) disk-like ZnO, CdS@ZnO-1 [61].

Significant inorganic synthesis subfields include solvothermal and hydrothermal preparations. The hydrothermal process serves as the foundation for an enhanced solvothermal approach [74]. The terms “hydrothermal” and “solvothermal” refer to the preparation via chemical reactions in an aqueous solution above the boiling point of water and non-aqueous solution at fairly high temperatures, respectively. The solvothermal technique differs from the other two procedures in that it uses an organic solvent instead of water as the solvent. Liu et al. developed a core–shell ZnO/CdS heterostructure for better photocatalytic H_2_ production [75]. Figure 5 illustrates the SEM micrographs of ZnO and CdS@ZnO with different conditions. In summary, solvothermal synthesis has several advantages for the preparation of ZnO/CdS heterostructure nanocomposites, including controlled size and morphology, high purity, enhanced photocatalytic activity, versatility, and easy scalability. However, it also has some potential disadvantages, including high temperature and pressure requirements, use of hazardous solvents, long synthesis time, difficulty in controlling stoichiometry, and high energy consumption.

### 2.5. Successive Ionic Layer Adsorption-Reaction (SILAR) Method

**Figure 6:**
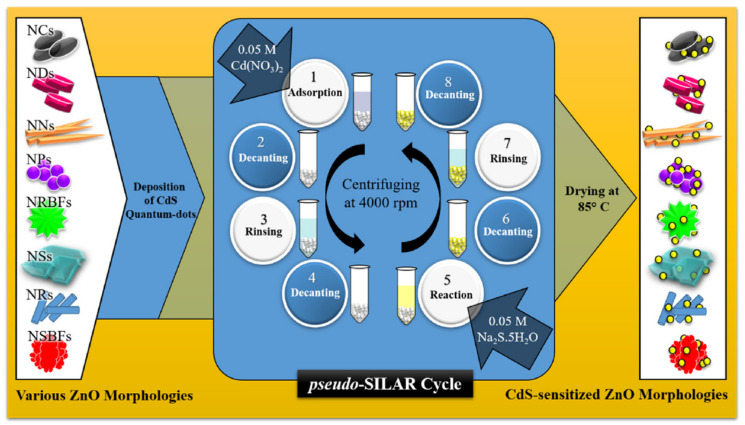
Schematic representation of Pseudo-SILAR method for depositing CdS quantum dots (QDs) on various ZnO morphologies [79].

The SILAR method, which is based on submerging the substrate under separately positioned cations and anions, is one of the chemical processes for creating uniform, large-area thin films in a well-ordered fashion. The SILAR approach has advantages over the existing fabrication techniques in terms of ease of use, low cost, and speed of reaction. In recent years, this technique has emerged as one of the most efficient ways to deposit a variety of materials [76,77,78]. Preparatory parameters can also readily control the grain size, film shape, and thickness of the depositing substance. Different kinds of conducting and non-conducting substrates can be used using this technique. Khan et al. synthesized ZnO/CdS heterostructures by using various ZnO morphologies [79]. Lai et al. fabricated a well-ordered nanorod-shaped ZnO/CdS thin film by the SILAR method and found a highest photocurrent density of 7.8 mA/cm^2^ [78]. Kolaei et al. synthesized a ZnO-CdS photoanode for enhanced water-splitting characteristics [63]. The SILAR method provides better morphology control with enhanced photoelectrochemical characteristics. A schematic representation of the pseudo-SILAR method for depositing CdS quantum dots (QDs) on various ZnO morphologies is illustrated in Figure 6. Overall, the SILAR method has several advantages for the synthesis of ZnO/CdS heterostructure nanocomposites, including low cost, good control over film thickness, large surface area, high purity, and room temperature synthesis. However, it also has some potential disadvantages, including slow deposition rate, limited film thickness, poor control over composition, difficulty in achieving uniformity, and limited applicability to more complex nanocomposites.

**Table 1:** Summary of the ZnO/CdS heterostructure fabrication processes.

Catalyst	Main Precursors		Method	Temperature	Reaction Time	Morphology	Ref
	Zinc	Cadmium					
CdS@ZnO	Zn(NO_3_)_2_ 6H_2_O	Cd(CH_3_COO)_2_ 2H_2_O	Atomic layer	250 °C	-	Nanospheres	[65]
ZnO/CdS	Zn(CH_3_COO)_2_·2H_2_O	Cd(NO_3_)_2_ 4H_2_O	Chemical bath deposition	60 °C	12 h	Nanofibers	[64]
ZnO/CdS	Zn(NO_3_)_2_ 6H_2_O	CdSO_4_	Chemical bath deposition	70 °C	-	Nanorods	[80]
ZnO/CdS/CuS	Zn(CH_3_COO)_2_·2H_2_O	CdCl_2_ 2H_2_O	Chemical solution deposition	120 °C	4 h	-	[81]
CdS/ZnO	Zn(CH_3_COO)_2_·2H_2_O	Cd(NO_3_)_2_ 4H_2_O	Hydrothermal	300 °C	3 h	Nanosheet	[82]
ZnO/CdS	ZnCl_2_	Cd(NO_3_)_2_ 4H_2_O	Hydrothermal	200 °C	24 h	Nanoparticles	[38]
ZnO/CdS	Zn(NO_3_)_2_ 6H_2_O	Cd(NO_3_)_2_ 4H_2_O	Hydrothermal	120 °C	12 h	-	[72]
ZnO/CdS	Zn(CH_3_COO)_2_·2H_2_O	CdCl_2_	Hydrothermal	40 °C	20 min	Nanosheets	[83]
ZnO/CdS	Zn(NO_3_)_2_ 6H_2_O	Cd(CH_3_COO)_2_ 2H_2_O	Hydrothermal	120 °C	24 h	-	[60]
ZnO/CdS	Zn(NO_3_)_2_ 6H_2_O	CdCl_2_ 5H_2_O	In situ	90 °C	1.5 h	Nanorods	[84]
CdS/ZnO	Zn(NO_3_)_2_	Cd(CH_3_COO)_2_ 2H_2_O	Microwave	-	20 min	Nanorods	[69]
ZnO/CdS	Zn(CH_3_COO)_2_·2H_2_O	Cd(CH_3_COO)_2_ 2H_2_O	Microwave	100 °C	2 h	Nanosheets	[59]
GO/CdS/ZnO	Zn(NO_3_)_2_ 6H_2_O	Cd(CH_3_COO)_2_ 2H_2_O	Microwave-assisted co-precipitation	240 W	30 min	Nanorods	[58]
ZnO/CdS	Zn(NO_3_)_2_ 6H_2_O	Cd(CH_3_COO)_2_ 2H_2_O	One-pot	RT	-	Nanostructures	[85]
CdS/ZnO	Zn(CH_3_COO)_2_·2H_2_O	Cd(NO_3_)_2_ 4H_2_O	Photodeposition technique	RT	30 min	Nanorods	[73]
CdS/ZnO	Zn(CH_3_COO)_2_·2H_2_O	Cd(CH_3_COO)_2_ 2H_2_O	Precipitation	90 °C	1 h	Nanoflowers	[37]
ZnO/CdS	Zn(NO_3_)_2_	Cd(NO_3_)_2_	Silar	250 °C	10 min	Nanorods	[76]
PbS/CdS/ZnO	Zn(CH_3_COO)_2_·2H_2_O	CdCl_2_	Silar	110 °C	4 h	Nanowire	[77]
ZnO/CdS/CdSe	Zn(NO_3_)_2_ 6H_2_O	Cd(NO_3_)_2_ 4H_2_O	Silar	RT	-	Nanorods	[86]
ZnO/CdS	Zn(CH_3_COO)_2_·2H_2_O	CdCl_2_ 5H_2_O	Silar	500 °C	2 h	Nanofilm	[78]
ZnO-CdS	Zn(CH_3_COO)_2_	Cd(NO_3_)_2_	Silar	300 °C	2 h	Nanorods	[63]
CdS@ZnO	Zn(CH_3_COO)_2_·2H_2_O	Cd(CH_3_COO)_2_ 2H_2_O	Solvothermal	80 °C	48 h	-	[61]
ZnO/CdS	Zn(CH_3_COO)_2_·2H_2_O	Cd(CH_3_COO)_2_ 2H_2_O	Ultra-sonication	80 °C	2 h	Nanostructures	[62]
ZnO-CdS	Zn(NO_3_)_2_ 6H_2_O	Cd(NO_3_)_2_ 4H_2_O	Wet chemical	100 °C	2 h	Nanoflower	[50]
ZnO-CdS	Zn(CH_3_COO)_2_·2H_2_O	Cd(CH_3_COO)_2_ 2H_2_O	Wet chemical coprecipitation	RT	2 h	Polycrystalline	[66]

No matter the synthesis technique employed to create ZnO/CdS heterostructures, it is hoped to minimize agglomeration. Additionally, it is claimed that ZnO/CdS heterostructures with an improved crystallinity, lower particle sizes, and controlled shape can be created using the hydrothermal method. Additionally, the nanoparticles are evenly spread and the agglomeration is successfully decreased. The microwave irradiation method offers a well-controlled size and morphology. Moreover, the SILAR approach is preferable for creating binary or ternary heterostructures with significant surface areas and well-ordered morphologies.

The choice of the synthesis method for ZnO/CdS heterostructure nanocomposites for enhanced photocatalytic applications depends on several factors such as the desired properties of the nanocomposites, the cost of the synthesis method, and the availability of the required equipment and materials. However, among the commonly used synthesis methods, the solvothermal and hydrothermal methods are more suitable for the synthesis of ZnO/CdS nanocomposites for enhanced photocatalytic applications. The solvothermal method offers advantages such as the ability to control the size, morphology, and composition of the nanocomposites, as well as high purity and crystallinity. This method also allows for the preparation of nanocomposites with a high surface area and efficient charge separation, which is crucial for enhanced photocatalytic performance. Similarly, the hydrothermal method offers advantages such as ease of synthesis, low cost, and the ability to synthesize nanocomposites with good crystallinity and controlled morphology. The hydrothermal method can also produce nanocomposites with a high surface area and efficient charge separation, making them suitable for enhanced photocatalytic applications.

In summary, both the solvothermal and hydrothermal methods have their advantages and can be suitable for the synthesis of ZnO/CdS heterostructures for enhanced photocatalytic applications. The choice of the method will depend on the specific requirements of the application and the availability of the required materials and equipment. Table 2 presents the advantages and disadvantages of different synthesis methods for ZnO/CdS heterostructures.

The preparation method of ZnO/CdS heterostructure nanocomposites can have a significant impact on their electronic structure, bandgap, adsorption properties, and photocatalytic performance. Different methods can lead to variations in the morphology, crystallinity, interface quality, and doping of the nanocomposites, which in turn affect their properties.

The preparation method influences the morphology and crystallinity of the nanocomposites. These structural variations can influence the electronic structure and bandgap of the nanocomposites. The interface between ZnO and CdS in the heterostructure is critical for charge separation and transfer. The preparation method can affect the interfacial quality by controlling the degree of lattice matching, interfacial defects, and surface states. A well-aligned and defect-free interface facilitates and enhances the charge transfer. The bandgap of ZnO/CdS nanocomposites can be tuned by varying the composition, size, and morphology. The preparation method influences these factors, thus affecting the bandgap and light absorption properties of the nanocomposites. Narrowing the bandgap can boost visible-light absorption and enlarge the photocatalytic performance. The surface area, porosity, and presence of defects or dopants can influence the adsorption properties of ZnO/CdS nanocomposites. The preparation method can control these factors, affecting the adsorption capacity and affinity toward the target molecules or pollutants. This, in turn, can influence the photocatalytic degradation or reaction kinetics. The combined effects of the above factors ultimately determine the photocatalytic performance of ZnO/CdS nanocomposites. Optimized preparation methods can lead to enhanced charge separation, efficient charge transfer at the interface, extended light absorption, and improved catalytic activity for various applications such as pollutant degradation, water splitting, or organic synthesis. It is important to note that the specific details of how each preparation method affects the properties of ZnO/CdS nanocomposites can vary depending on the experimental conditions, parameters, and techniques employed. Therefore, it is essential to refer to the relevant literature or specific research studies for a comprehensive understanding of the impact of a particular preparation method on the nanocomposite properties.

## 3. Morphology Control of ZnO/CdS Heterostructures

In order to enhance the efficiency of ZnO/CdS heterostructure photocatalysts, some approaches have been used, viz., morphology control, bandgap engineering, and heterostructure formation. The morphology of the heterostructure photocatalysts plays a vital role in their characteristics. Hence, the morphology control is predominantly significant for ZnO/CdS heterostructure photocatalysts. The anticipated photocatalytic effectiveness and stability can be attained by altering the morphology of heterostructures by forming binary and ternary heterostructures. The previous studies demonstrated that nanorod-based heterostructures (1D/0D) showed the highest photocatalytic performance. This may be because they have a greater aspect ratio, which diminishes the rate of the electron–hole recombination. These findings imply that the photocatalysis and optical properties of heterostructures are significantly influenced by their size and shape. In order to create heterostructures with increased photocatalytic activity, it is crucial to modify the morphology of the photocatalysts.

The morphology of ZnO/CdS heterostructures plays a crucial role in determining their photocatalytic performance. The heterostructure morphology refers to the arrangement, size, and shape of the ZnO and CdS components within the composite material. Particularly, there are a few key factors, such as an increased surface area, light harvesting capacity, diffusion of reactants, defects, reactive sites, charge separation and transport, in which the morphology influences the photocatalytic performance of the ZnO/CdS heterostructures. By optimizing the morphology, one can enhance the catalytic activity and efficiency of the heterostructure for various photocatalytic applications.

### 3.1. Binary Heterostructured Photocatalysts

Among the numerous approaches, the development of heterostructures has been evidenced as an efficient strategy for improved photocatalytic performance than individual components. After the development of semiconductor heterostructures, band alignment occurs when n-type or p-type semiconductors with different bandgaps are combined. Because of the inherent potential at the semiconductor–semiconductor interface, which can facilitate the separation and migration of photoinduced e^−^–h^+^ pairs, semiconductor heterostructures are a useful architecture for enhancing photocatalytic activity. In general, for effective and stable photocatalysts, the surface characteristics, crystal structure, material selection, and construction of heterostructure photocatalysts need to be carefully taken into account [87]. Effective charge separations are supported by the development of semiconductor heterostructures. ZnO/CdS heterostructures with a different morphology have been successfully created by numerous researchers using a variety of techniques.

**Figure 7:**
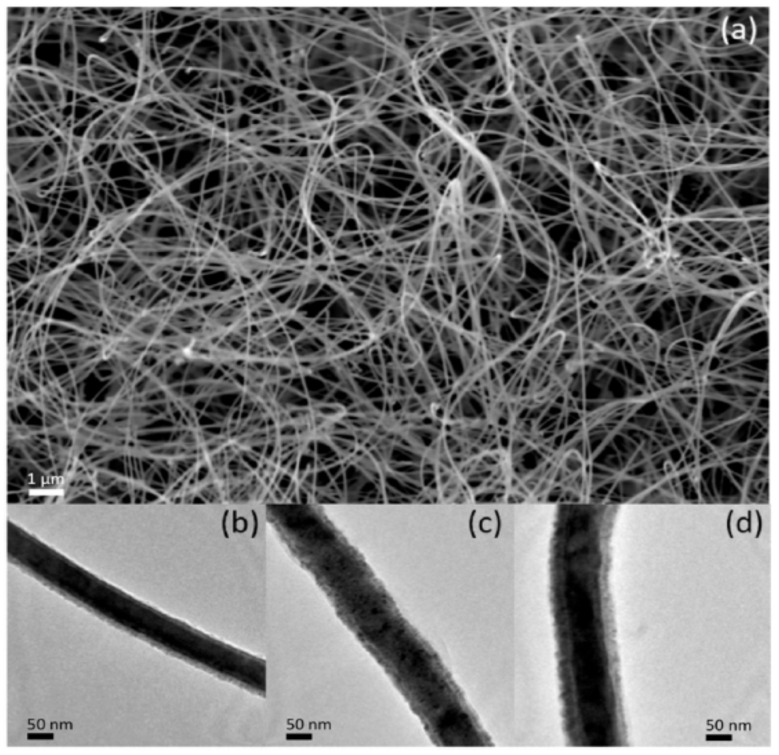
(**a**) SEM, (**b**–**d**) TEM micrographs of CdS-ZnO core–shell nanowires [87].

**Figure 8:**
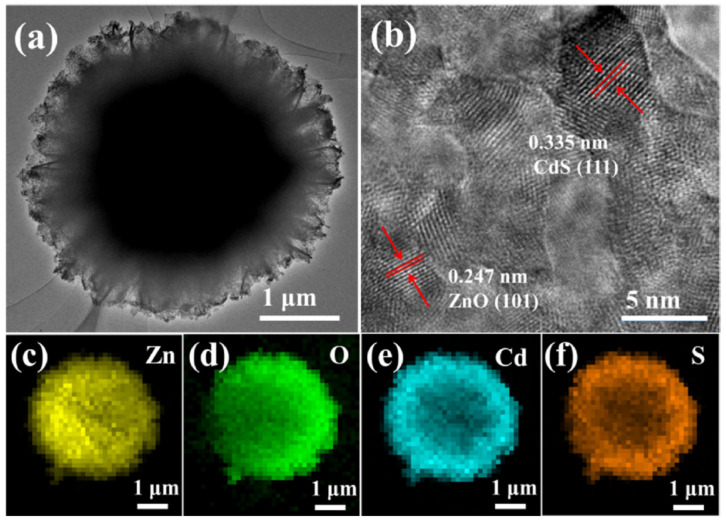
(**a**) TEM, (**b**) HRTEM, and (**c**–**f**) EDS mapping images of ZnO/CdS heterostructure [83].

Saxena et al. reported that ZnO-CdS nanostructures fabricated by the coprecipitation method showed a maximum photodegradation activity of 99% in 110 min over MB dye, which surpasses that of pure ZnO and CdS by a wide margin [66]. According to Gurugubelli et al., ZnO/CdS heterostructures synthesized by the chemical precipitation method showed a highest RhB dye degradation efficiency of 98.16% in 80 min [40]. The bandgap energy (2.9796 eV) was also well tuned by the heterostructure formation. Tso et al. synthesized CdS-ZnO core–shell nanowires by the RF sputtering method. The XRD study revealed the hexagonal phase for both ZnO and CdS. A highest photocatalytic hydrogen production rate of 9618 mmol/g/h was recorded [87]. Figure 7 depicts the SEM (a) and TEM (b–d) micrographs of CdS-ZnO core–shell nanowires. Utilizing a microwave preparation technique, Huo and Chen created a CdS/ZnO heterostructure that significantly increased hydrogen production efficiency [69]. The noticeable improvement in the H_2_ production rate could be attributed to the reduced bandgap energy of 2.62 eV. Revathi et al. utilized the microwave technique to fabricate ZnO/CdS heterostructures for improved photocatalytic hydrogen production [59]. Senasu et al. synthesized a ZnO/CdS photocatalyst with a very high electron–hole separation efficiency and good visible-light harvesting capability [60]. Bai et al. fabricated CdS/ZnO heterostructures by the wet chemical method for efficient Cr removal and MB decolorization under visible light [88]. Enhanced photoelectrochemical performance was achieved by a hydrothermally fabricated ZnO/CdS heterostructure thin film by Xu et al. [89]. A direct Z-scheme ZnO/CdS heterostructure was developed by Wang et al., which exhibited an improved hydrogen production rate of 4134 μm/g/h [83]. The flower-like TEM (a), HRTEM (b), and (c–f) EDS mapping images of the ZnO/CdS heterostructure are displayed in Figure 8. The reported literature demonstrated that the enhanced photocatalytic performance may be ascribed to the significantly improved visible-light absorption capacity, and photoinduced charge carrier separation and transfer caused by the strong heterostructure formation at the interface of the ZnO and CdS nanostructures. A detailed analysis of recent progress on the photocatalytic capabilities of ZnO/CdS heterostructures is presented in Table 3.

Binary ZnO/CdS heterostructures offer several advantages over individual ZnO and CdS for photocatalysis. Here are some possible reasons: (i) Enhanced photocatalytic activity: ZnO and CdS have different bandgap energies, which means they absorb different wavelengths of light. By combining them into a heterostructure, the absorption range of the material can be expanded, allowing for a more efficient use of solar energy. This can lead to a greater photocatalytic performance compared to the individual components. (ii) Reduced electron–hole recombination: In photocatalysis, electron–hole recombination is a key problem that reduces the efficiency of the process. In a binary ZnO/CdS heterostructure, the different bandgap energies of the two materials can create a type-II heterojunction, which can enable the separation of photoinduced electron–hole pairs and reduce recombination. This results in a higher efficiency and better photocatalytic performance. (iii) Improved stability: ZnO and CdS are both susceptible to photo-corrosion under certain conditions. However, in a binary ZnO/CdS heterostructure, the two materials can protect each other from photo-corrosion by forming a stable interface. This can increase the overall stability and longevity of the photocatalyst. (iv) Tunable properties: The properties of a binary ZnO/CdS heterostructure can be tuned by adjusting the relative amounts of the two materials, the size and shape of the nanoparticles, and the preparation method. This can allow for the optimization of the photocatalytic properties for specific applications. Overall, binary ZnO/CdS heterostructures offer a promising approach for improving the photocatalytic activity and stability of these materials and have potential applications in a range of fields including environmental remedies, energy storage, and optoelectronics.

**Table 3:** Detailed report of current study on the photocatalytic capabilities of ZnO/CdS heterostructures.

Photocatalyst	Type of Heterostructure	Bandgap	Pollutant	Dosage	Light Source	Efficiency	Ref
ZnO/CdS	--	2.4 eV	MB	--	UV–visible	99% in 110 min	[66]
ZnO/CdS	Type-II	3.05 eV	RhB	5 mg/60 mL	Visible	72.4% in 120 min	[81]
			MO			88.5% in 120 min	
ZnO/CdS	--	3.37 eV	RhB	5 mg/20 mL	UV–visible	90% in 80 min	[50]
CdS/ZnO	--	1.78 eV	RhB	15 mg/L	UV–visible	91.5% in 150 min	[90]
CdS/ZnO	Type-II	3.04 eV	RhB	-	Visible	85% in 30 min	[73]
RGO/ZnO/CdS	--	--	Aqueous chromium	20 mg/L	Visible	93.2% in 30 min	[72]
ZnO/CdS	Hierarchical	-	Bisphenol—A	25 mg/50 mL	Visible	55% 30 min	[64]
					Visible	100% 30 min	
ZnO/CdS	Core–shell	-	H_2_ production	100 mg/200 mL	Visible	6.696 mmol/g/h	[38]
CdS/ZnO	Core–shell	2.68 eV	H_2_ production	20 mg/80 mL	Visible	7.94 mmol/g/h	[61]
ZnO/CdS	Z-scheme	-	H_2_ production	50 mg/80 mL	Visible	4134 μmol/g/h	[83]
CdS/ZnO	--	2.62 eV	H_2_ production	50 mg/100 mL	Visible	4076 μmol/g/h	[69]
ZnO/CdS	Z-scheme	2.81 eV	H_2_ production	20 mg	Solar	1545 ± 0.3 μmol/g/h	[59]
CdS/ZnO	Core–shell	2.4 eV	H_2_ production	10 mg/100 mL	Visible	11.13 mmol/g/h	[65]

### 3.2. Ternary Heterostructured Photocatalysts

In order to create more contact surfaces and create a new transfer channel for photoinduced charge carriers, it is also possible to create a ternary heterostructure by linking a new semiconductor component with the ZnO/CdS nanocomposite. This allows for the realization of multilevel charge transport. Depending on how well the energy levels of the various semiconductors match, either all three semiconductors in the ternary heterostructure contact the other two to form a heterojunction interface, or only some of the components do. Numerous studies have reported using ZnO/CdS-based ternary heterostructures as an effective photocatalyst to overcome the limitations of bare and binary heterostructures. This has led to better optical properties, interfacial charge transfer efficiency, and photodegradation activity of many dyes and orgonic compounds from wastewater under visible or solar light. Recent reports have revealed that the photocatalytic H_2_ production rate was also increased by the development of ternary compounds. The photocatalytic activity of ZnO/CdS-based ternary heterostructures is summarized in this section. Numerous investigations have concentrated on binary ZnO/CdS nanocomposites for enhanced visible-light photocatalysis so far, as discussed in the preceding section. Researchers have focused on ternary compounds in addition to binary heterostructures to create new photocatalysts with excellent photocatalytic activity under visible-light and solar-light irradiation. In order to create ternary heterostructures such as GO/CdS/ZnO, ZnO/Zn_x_Cd_1−x_S/CdS, ZnO-ZnS-CdS, ZnO/CdS/CuS, CdS/ZnO/TiO_2_, PbS/CdS/ZnO, ZnO/CdS/CdSe, CdS@ZnO/g-C_3_N_4_, ZnO–CdS–Cu, Al-ZnO/CdS/TiO_2_, MoSe_2_-CdS-ZnO, CdSe/CdS/ZnO, ZnO/CdS/MoS_2_, ZnO/CdS/MoS_2_, ZnO@ZnFe_2_O_4_/CdS, CdS/ZnS/ZnO, CdSe/CdS@ZnO, ZnO/CdS/GO, CdS@ZnSeZnO, CdS/g-C_3_N_4_/ZnO, CdS/ZnO/TiO_2_, and CuSe-ZnO/rGO/CdS, ZnO/CdS was combined with a variety of co-sensitizers [81,86,91,92,93,94,95,96,97,98,99,100,101,102,103,104,105,106,107,108]. A detailed analysis of recent progress on the photocatalytic capabilities of ZnO/CdS-based ternary heterostructures is presented in Table 4.

**Figure 9:**
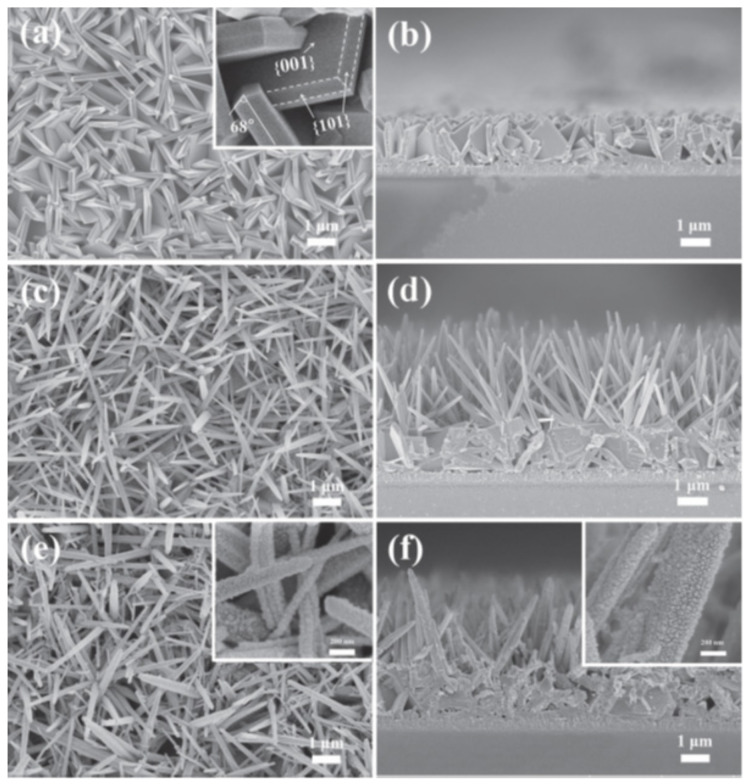
SEM micrographs of TiO_2_ (**a**) top, (**b**) cross-sectional view, ZnO/TiO_2_ (**c**) top, (**d**) cross-sectional view, and CdS/ZnO/TiO_2_ ternary composite (**e**) top, (**f**) cross-sectional view [93].

In this class, a new ternary photocatalyst with improved physicochemical properties is produced by combining three semiconductors with dissimilar bandgaps. The separation of e^−^–h^+^ pairs is accelerated and the photocatalytic efficiency is increased in ternary compounds due to the variance in the energy levels for the CB of the united semiconductors, which forces a quick transfer of photoinduced charge carriers from one semiconductor to another adjacent semiconductor in cascade form [109]. According to Chen and Huo, the photocatalytic H_2_ evolution rate of CdS/ZnO binary heterostructures was significantly improved after being combined with graphene oxide (GO), which might be ascribed to the increased specific surface area, diminished energy bandgap, and rapid photoinduced charge carriers’ migration rate [58]. Holi et al. described the fabrication of PbS/CdS/ZnO nanowire arrays using the SILAR method. They found that the rate of H_2_ generation with PbS/CdS/ZnO is 1.5 and 6 times more than that of binary CdS/ZnO and bare ZnO nanowire arrays [77]. An extreme photocurrent density of 12.1 mA/cm^2^ was recorded by the ternary CdS/ZnO/TiO_2_ ternary heterostructure, which is almost 27 times more than the bare TiO_2_ [93]. Figure 9 displays the SEM micrographs of the top view and cross-sectional view of TiO_2_ (a–b), ZnO/TiO_2_ (c–d), and CdS/ZnO/TiO_2_ ternary composites (e-f).

**Figure 10:**
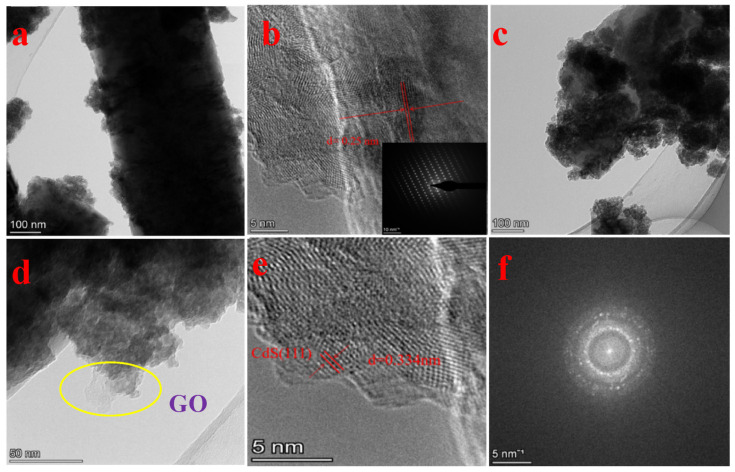
TEM images of 0.40GZC: (**a**–**e**) TEM, and (**f**) SAED [58].

Li et al. reported an extreme photocurrent density of 6.244 mA/cm^2^ for ZnO/CdS/CdSe heterostructure, which is 4.73 and 57.28 times that of CdS/CdSe and pure ZnO nanostructures, respectively [86]. According to Nandi and Das, the ZnO/CdS/CuS ternary heterostructure exhibited a superior photodegradation efficiency than binary ZnO/CdS and pristine ZnO [81]. The improvement in photodegradation efficiency over RhB and MO dye could be ascribed to the diminished charge carrier recombination rate and bandgap energy. The bandgap energy of the ZnO/CdS/CuS ternary heterostructure is around 2.97 eV, which also significantly improves the catalytic activity by increased charge separation and transfer. Hashem et al. synthesized a ZnO/CdS/g-C_3_N_4_ ternary heterostructure by microwave-assisted synthesis and achieved excellent photodegradation over RhB dye, which is very much higher than the binary heterostructure and bare nanostructures [94]. Zhou et al. testified an excellent photodegradation and H_2_ generation capacity of a uniformly distributed and spherical-shaped CdS/ZnS/ZnO ternary heterostructure [105]. The highest efficiency of the ternary composite could be ascribed to the noticeable change in the specific surface area, average pore volume, and visible-light absorption capacity. According to Liu et al., the CdS/g-C_3_N_4_/ZnO heterostructures showed a better photocatalytic activity than the binary ZnO/CdS, g-C_3_N_4_/ZnO, and pure ZnO [106]. Wang et al. achieved the high photocatalytic H_2_ evolution statistics 1073 μmol/g/h using CuS-ZnO/rGO/CdS heterostructures under visible-light illumination [108]. Sun et al. described that the photocatalytic hydrogen generation rate was much improved in optimized CdS/ZnS/ZnO (CZZ-4) ternary heterostructures related to the pure ZnO and ZnO/CdS binary heterostructures [102]. It was also noticed that the PEC performance of ternary CZZ-4 heterostructures was 2.28 and 93.54 times more advanced than that of binary ZnO/CdS and bare ZnO. According to Tang et al., the ternary ZnO/CdS/MoS_2_ nanostructures exhibited an extreme photocurrent density of 20.32 mA/cm^2^. Moreover, more than 94% of the amoxicillin was degraded within 60 min under visible-light illumination [100]. Similarly, Jia et al. described that ZnO/CdS/MoS_2_ heterostructures exhibited an outstanding H_2_ production rate of 10247.4 μmol/g/h which is almost three times superior than binary ZnO/CdS and thirty times larger than pristine CdS [99]. Figure 10 displays the (a–e) TEM micrographs and (d) SAED patterns of 0.40GZC.

Ternary ZnO/CdS-based heterostructures offer several advantages over binary ZnO/CdS heterostructures for photocatalysis. Here are some potential benefits: (i) Enhanced photocatalytic activity: The incorporation of a third component in the ternary ZnO/CdS heterostructure can further extend the light absorption range and create new synergistic effects among the different materials, which leads to a substantial improvement in photocatalytic activity compared to binary ZnO/CdS heterostructures. (ii) Improved charge separation and transfer: The addition of a third component in the ternary ZnO/CdS heterostructure can provide additional energy levels that facilitate efficient charge separation and transfer. This can reduce charge recombination and increase the number of active sites involved in the photocatalysis process. (iii) Better stability and durability: The introduction of a third component can also improve the stability and durability of the ternary heterostructure, by reducing the photo-corrosion and degradation of ZnO and CdS. The third component can act as a protective layer and enhance the overall stability of the photocatalyst. (iv) Versatility and customization: Ternary ZnO/CdS-based heterostructures offer greater versatility in terms of composition and structure design, allowing for the customization of the photocatalytic properties for specific applications. The choice of the third component can be tailored to optimize the photocatalytic performance and target specific reaction pathways or pollutants. (v) Expanded range of applications: The introduction of a third component in the ternary ZnO/CdS heterostructure can enable the exploration of new photocatalytic reactions and expand the range of applications. The combination of multiple materials with complementary properties can unlock novel photocatalytic processes, such as the degradation of specific pollutants or the activation of specific reactants. Overall, ternary ZnO/CdS-based heterostructures offer promising prospects for enhancing photocatalytic performance, improving stability and durability, and expanding the range of applications. However, it is important to note that the specific benefits and performance of ternary heterostructures can diverge dependent on the selection of the third component and the overall design of the heterostructure. Moreover, different types of ZnO/CdS heterostructures, namely p-n, Type-I, Type-II, Type-III, Z-scheme, and S-scheme, exhibit distinct characteristics and functionalities. The specific performance and limitations of ZnO/CdS heterostructures may vary based on fabrication techniques, material quality, and application requirements. Table 5 demonstrates the advantages and disadvantages of each type of heterostructure.

**Table 4:** Detailed report of recent progress on the photocatalytic capabilities of ZnO/CdS-based ternary heterostructures.

Photocatalyst	Type of Heterostructure	Bandgap	Pollutant	Dosage	Light Source	Efficiency	Ref
GO/ZnO/CdS	--	2.34 eV	H_2_ production	0.40 g/L	Visible	6511 μmol/g/h	[58]
CdS/ZnO/TiO_2_	Hierarchical	2.34 eV	Nitrite in water	3%	Visible	92.58%	[93]
ZnO/CdS/CdSe	--	1.754	--	--	Visible	6.244 mA/cm^2^	[86]
ZnO/CdS/CuS	--	2.97	RhB	5 mg/60 mL	Visible	82%	[81]
CuSeZnO/rGO/CdS	--	2.2 eV	Hydrogen evolution	1 g	Visible	1073 mmol/g/h	[108]
MoSe_2_-CdS-ZnO	Z-scheme	--	Hydrogen evolution	--	Visible	116.4 μmol/cm^2^	[97]
ZnO@ZnFe_2_O_4_/CdS	Hierarchical	--	CO_2_ reduction	5 mg	Visible	95.84 μmol/g/h	[101]
ZnO/CdS/MoS_2_	--	2.24 eV	Amoxicillin	--	Visible	94% in 60 min	[100]
ZnO/CdS/MoS_2_	S-scheme	--	Hydrogen evolution	15 mg	Visible	10,247.4 μmol/g/h	[99]
CdS/ZnS/ZnO	--	2.4 eV	Hydrogen evolution	10 mg	Visible	51.45 mmol//g/h	[102]

## 4. Photocatalytic Mechanism and Applications

**Figure 11:**
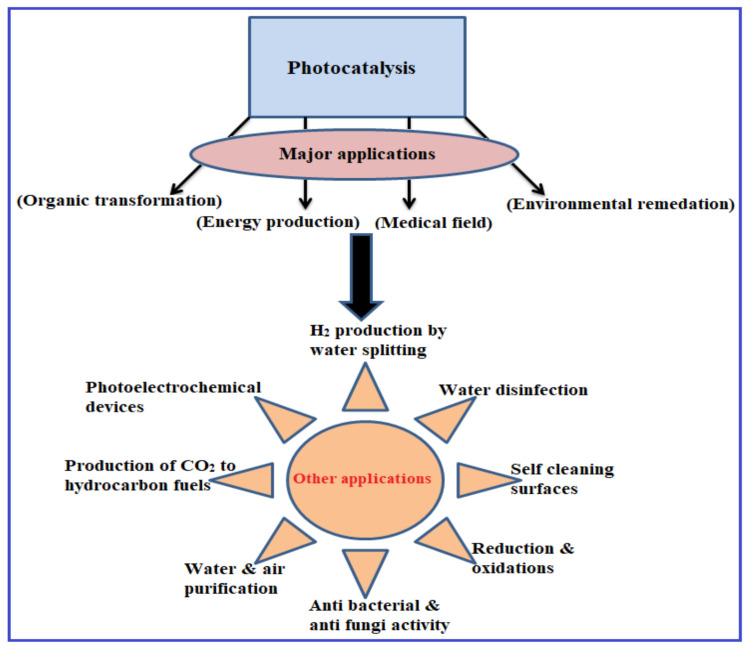
Detailed applications of the photocatalysis technique.

Photocatalysis is a fantastic tool for converting energy and cleaning up the environment. Here is a summary of the potential photocatalytic mechanisms and uses of ZnO/CdS heterostructures. In a typical process, the photons transcend the bandgap of metal oxide semiconductors to generate e^−^–h^+^ pairs within the particle. The photoinduced e^−^–h^+^ pair is the intermediate free radical that results from this process. Shortly after being created, most charge carriers in the semiconductor go through non-radiative electron–hole pair recombination. The remaining charge carriers are trapped in bandgap states such as shallow electron traps, deep electron traps, and hole (h^+^) traps. However, the concept of photocatalysis can be used in various sectors and for different practical applications. The diagram pertaining to the various applications of photocatalysis is presented in Figure 11.

The intermediate species, “electron–hole” pair is created when the bandgap is excited. Surface imperfections and appropriate charge carrier scavengers play a key role in improving redox processes by lowering the intensity of electron–hole recombination [110]. The holes in the VB are influential oxidants, while the electrons in the CB are authoritative reluctant. Directly or indirectly, the oxidation potential of holes affects the photodegradation process. Conduction band electrons are also responsible for producing some of the reactive oxygen species needed for photodegradation. In addition to that, some other free radicals such as oxides, hydroxyl, and other non-harmful degradation products are released [111]. The valance electrons are excited to the conduction band by the crossing energy bandgap due to the incidental photon energy (hυ), and further oxidation and reduction reactions took place to obtain the CO_2_ and H_2_O as a non-harmful degradation output. The photocatalytic degradation mechanism steps for the removal of organic dyes under UV/Vis-light illumination, generation of free radicals, and non-harmful degradation products are prescribed below:

ZnO + hυ (incident light) → h^+^ + e^−^

O_2_ + e^−^ → O_2_^•−^

H_2_O → OH^−^ + H^+^

O_2_^•−^ + H^+^ → HOO^•−^

HOO^•−^ + e^−^ → HO_2_^•−^

HOO^•−^ + H^+^ → H_2_O_2_

H_2_O_2_ + e^−^ → OH^•−^ + OH^−^

H_2_O + h^+^ → H^+^ + OH^•^

h^+^ (VB) + OH^−^ → OH^•^ (Hydroxyl ions)

O_2_ + e^−^ (CB) → O2^•−^ (Super oxide ions)

Pollutants + ZnO + OH^•^ + O_2_^•−^ → ZnO + CO_2_ + H_2_O (non-harmful products)

**Figure 12:**
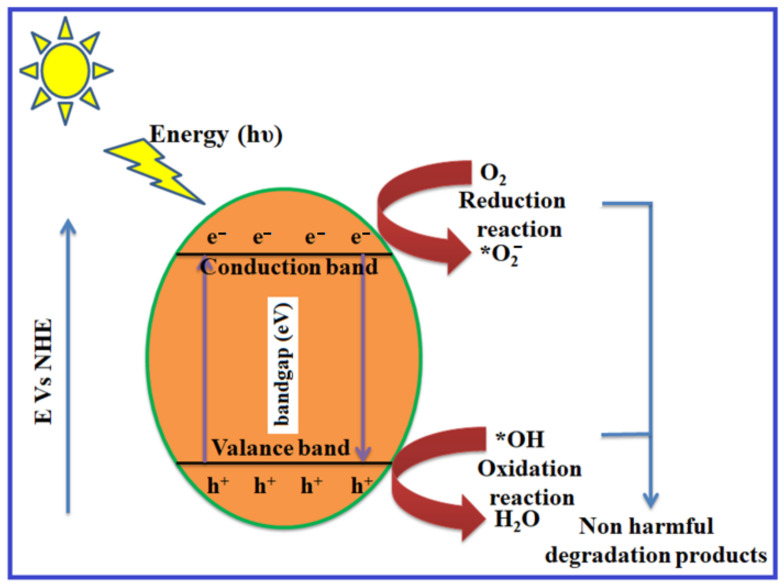
General Photodegradation mechanism on the surface of ZnO.

Even though ZnO acts as good photocatalyst material, it has some demerits, such as its working capacity under UV-light illumination rather than visible light. The rate of recombination of photoinduced charge carriers and corrosion by the action of light (photocorrosion) seem to be other negative factors. To overcome this hindrance, the process of doping with a variety of groups of metal ions into the ZnO host is preferred, as it enhances the photocatalytic degradation efficiency of the catalyst material [96]. The general photodegradation mechanism on the surface of the ZnO nano semiconductor is depicted in Figure 12.

### 4.1. Photodegradation Mechanism in Binary Heterostructures

**Figure 13:**
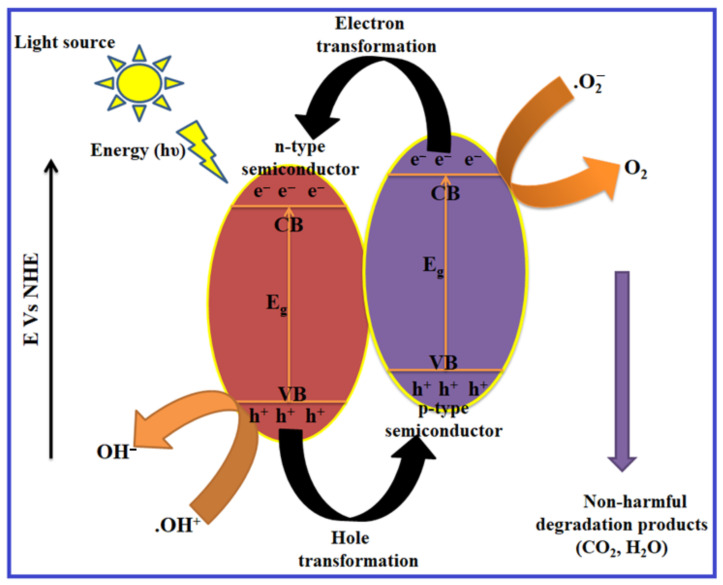
Charge transfer mechanism in binary heterostructures.

In a composite heterostructure, both semiconductors are encouraged to produce electron–hole pairs. A semiconductor with a lower conduction band may receive photogenerated electrons from a semiconductor with a higher CB. The higher valance band semiconductor (HVB) receives the photoinduced holes as they are being transferred from the lower VB semiconductor [112]. This could result in the reduced recombination of electrons and holes, which would improve photocatalytic efficiency. When a light source of a certain energy (hυ) is incident on the heterojunction semiconductor system, then electrons from the higher conduction band of p-type are transferred to the lower conduction band of the n-type material, and furthermore, consequently, holes from the lower valance band of the n-type semiconductor are migrated to the lower valance band of the p-type semiconductor. The migration of photoinduced charge carriers on the surface of the heterogeneous composite results in the establishment of uniform electric field at the interface between the p-type and n-type semiconductor. The proposed mechanism of the photoinduced charge carrier (e^−^–h^+^) transformation on the binary heterostructure is depicted in Figure 13.

**Figure 14:**
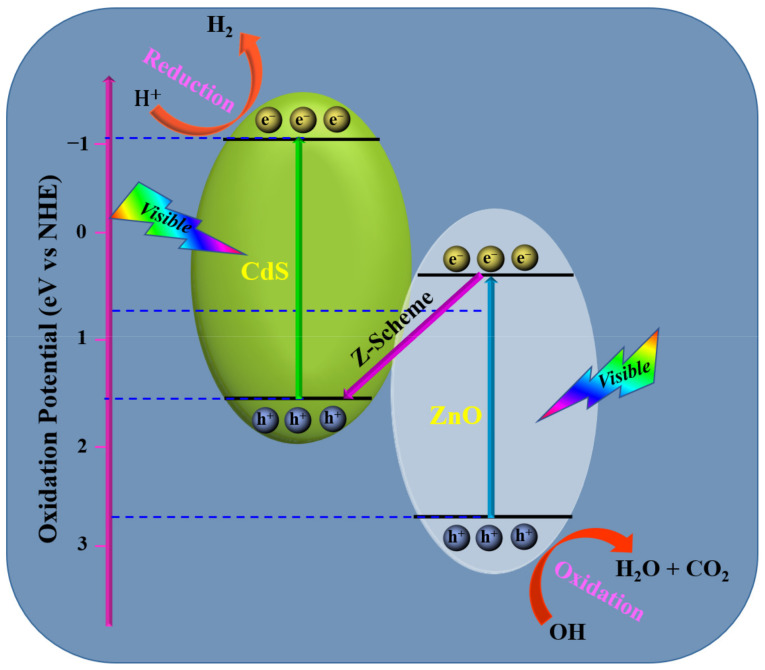
A direct Z-scheme photocatalytic mechanism for ZnO/CdS heterostructures.

In Figure 14, a direct Z-scheme photocatalysis process of the ZnO/CdS heterostructure is presented. A close contact is created when CdS is placed on the ZnO surface. Due to the greater negative Fermi level of the CdS, electrons will then flow from the CdS to ZnO via the close contact surface, leaving holes in the CdS. The Fermi levels did not equilibrate until the electron diffusion stopped. As a result, an internal electric field was generated at the contact between ZnO and CdS and was directed from CdS to ZnO. The internal electric field controlled the migration of photoinduced electrons from the CB of CdS to the CB of ZnO and photogenerated holes from the VB of ZnO to the VB of CdS during the photocatalytic reaction. In contrast, the photoexcited electrons remained in the CB of CdS and the photoexcited holes remained in the VB of ZnO due to the acceleration of the electron transfer along the Z direction from the CB of ZnO to the VB of CdS. Therefore, the separation of photoexcited charge carriers at the ZnO/CdS interface benefits from the growth of a direct Z-scheme photocatalyst. Additionally, the significant hole oxidization ability of ZnO and great electron reducibility of CdS are preserved, which enhances the photocatalytic H_2_ generation even more.

### 4.2. Photodegradation Mechanism in Ternary Heterostructures

To learn more about the functions of the superoxide anion radical (•O^2−^), hydroxyl radical (•OH), and photogenerated holes (h^+^) in the photocatalytic process, the generation mechanisms of reactive oxygen species (ROS) were investigated. Therefore, in a ternary system, h+ was crucial for the degradation of contaminants. The following is a description of the pollutant degradation process through photocatalysis:

Photocatalyst + hυ → h^+^ + e^−^

e^−^ + O_2_ → •O_2_^−^

O_2_ + 2H_2_O → 2H_2_O_2_

H_2_O_2_ + 2e^−^ → 2•OH

Pollutant + •O_2_^−^ → oxidation product

Pollutant + h^+^ → oxidation product

Pollutant + •OH → oxidation product

**Figure 15:**
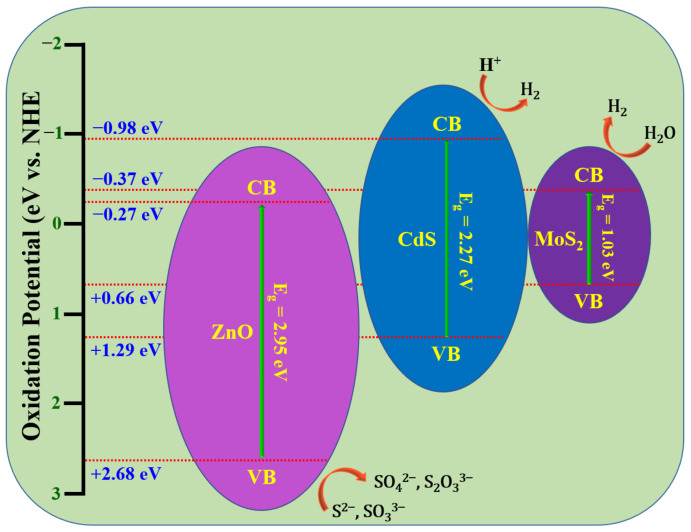
S-scheme mechanism in ZnO/CdS/MoS_2_ heterostructure.

Figure 15 suggests a putative S-scheme mechanism. The creation of a confined interface with an interior electric field and twisting band is caused by the same crystal structure, proper energy level site, and lower Fermi level of ZnO than CdS. Though ZnO cannot be stimulated due to the huge bandgap in visible light, CdS can produce electron–hole pairs. With the help of an electric field, photoinduced holes in CdS VB will swiftly move via the close interface, preventing the recombination of electron–hole pairs. When joined with a third substance (co-catalyst) to create a ternary nanocomposite, the ZnO/CdS heterostructures offer several advantages. A metal oxide, metal chalcogenide, or corban material can be linked with the ZnO/CdS binary heterostructure to increase the photocatalytic efficiency and stability of the catalyst. The strongly reductive electrons that had collected in the CB of CdS were immediately caught and disbursed by the co-catalyst upon its introduction. It is possible to significantly improve the separation of photoinduced electrons and holes in the ternary, leading to higher photocatalytic activity.

An overview of the literature speaks out regarding the diversity of morphological, bandgap characteristics, optimized composition, temperature, and synthetic condition, etc., to realize a semiconductor heterostructure with an excellent photocatalytic efficiency. In comparison with that for the cases of semiconductor/metal and semiconductor/insulator hetero junction composites, etc., the design of a ZnO-based p/n-type semiconductor heterojunction nanocomposite seems to be an effective form of approach to diminish the recombination and higher absorption in the vicinity of the visible range of the solar spectrum. The tunable nature of the bandgap of the semiconductor material is expected to usher a significant change in the photocatalytic activity (PCA) to anticipate an increase in the separation of photoinduced charges (electron–hole) generated on the surface for superior photocatalytic degradation efficiency. Hence, for the current investigation, the development of a ZnO/CdS (wide bandgap) heterostructure through selective band alignment techniques is planned that would realize a more efficient photocatalytic material, to degrade an organic dye, and H_2_ generation. However, the physical mechanism mediated by the production of free radical agents, viz., H^+^, •OH, HO_2_, H_2_O_2_, and •O_2_^−^ is expected to dominate the scenario. In the case of the possible matching configuration of the E-bands (VB and CB) of nanocomposites, the electrostatic potential, i.e., both the valence band and conduction bands of the p-type semiconductor, would be comparatively negative than that of ZnO at optimized compositions to drive higher visible-light-driven PCA. Hence, carriers are supposed to be transferred/made abundantly available in the conduction band of the shell material.

The photocatalytic mechanism of ZnO/CdS heterostructures can vary in acidic, basic, and neutral solutions due to differences in the pH-dependent surface chemistry and redox reactions. In an acidic solution, ZnO/CdS heterostructures exhibit specific characteristics such as the surface of ZnO is positively charged due to the protonation of hydroxyl groups (OH^−^) under acidic conditions. The photogenerated holes (h^+^) in ZnO can react with water (H_2_O) to generate hydroxyl radicals (•OH), which exhibit a strong oxidative power. CdS nanoparticles typically have a negative surface charge in acidic solutions. Under illumination, CdS generates electron–hole pairs (e^−^/h^+^). The photogenerated electrons (e^−^) can transfer to the CB of ZnO, while the photogenerated holes (h^+^) migrate to the CdS surface. The photogenerated electrons in ZnO and CdS can participate in reduction reactions, while the photoinduced holes in ZnO and hydroxyl radicals (•OH) can undergo oxidation reactions. Hydroxyl radicals (•OH) are produced from the reaction of photogenerated holes in ZnO with water (H_2_O). The generated ROS and photogenerated electrons participate in oxidative reactions, degrading organic pollutants or other target species.

In a basic solution, the surface chemistry and charge characteristics of ZnO/CdS heterostructures differ from those in an acidic solution. The surface of ZnO becomes negatively charged under basic conditions due to the deprotonation of surface hydroxyl groups (OH^−^). The photogenerated holes (h^+^) in ZnO can react with hydroxide ions (OH^−^) in the solution. CdS nanoparticles retain their negative surface charge in basic solutions. The photogenerated electrons (e^−^) transfer to the CB of ZnO, while the photogenerated holes (h^+^) migrate to the CdS surface. The photocatalytic mechanism in a basic solution generally follows similar steps as in an acidic solution, including charge separation, redox reactions, ROS generation, and pollutant degradation. However, the pH-dependent surface charge and the availability of hydroxide ions (OH^−^) can influence the kinetics and reaction pathways.

In a neutral solution, the pH-dependent surface charge of ZnO/CdS heterostructures is intermediate between acidic and basic conditions. The photocatalytic mechanism in a neutral solution can exhibit characteristics of both acidic and basic solutions. The surface charge of ZnO is relatively neutral in a pH solution. The photogenerated holes (h^+^) can react with water (H_2_O) to produce a combination of hydroxyl radicals (•OH) and hydroxide ions (OH^−^). The surface charge of CdS nanoparticles remains negative. The photogenerated electrons and holes can migrate to their respective regions due to the built-in electric field at the ZnO/CdS interface. The separated electrons and holes participate in redox reactions with surrounding species, such as adsorbed water or dissolved oxygen, leading to the formation of ROS. The ROS are responsible for the degradation of organic pollutants. It is important to note that the specific reactions and ROS involved in the photocatalytic process can change dependent on the type of organic pollutants and the specific experimental conditions.

In addition, free radicals play a crucial role in the photocatalytic activity of ZnO/CdS heterostructures. When ZnO and CdS absorb photons with energy equal to or greater than their bandgaps, electron–hole pairs (e^−^/h^+^) are generated. These photogenerated charge carriers can undergo recombination, which is a nonproductive process that reduces photocatalytic efficiency. Free radicals, on the other hand, can scavenge the photogenerated charge carriers and prevent recombination. This leads to an increase in the lifetime of the charge carriers and a corresponding enhancement in the photocatalytic activity of the heterostructures. The two most generated free radicals in the photocatalytic process of ZnO/CdS heterostructures are hydroxyl radicals (•OH) and superoxide radicals (•O_2_^−^). These radicals are highly reactive and can oxidize organic pollutants, leading to their degradation. The hydroxyl radicals are formed through the reaction of photogenerated holes with water or hydroxide ions, while superoxide radicals are formed through the reaction of photogenerated electrons with oxygen. In addition to scavenging charge carriers, free radicals can also participate in redox reactions with organic pollutants, leading to their degradation. For example, hydroxyl radicals can attack the carbon–carbon double bonds and aromatic rings in organic pollutants, leading to the formation of less complex and more soluble compounds that are easier to degrade. However, the presence of excess free radicals can also lead to side reactions, such as the oxidation of the photocatalyst itself, which can reduce its photocatalytic activity. Therefore, it is important to maintain an optimal concentration of free radicals in the photocatalytic process to achieve maximum efficiency. Overall, the generation and scavenging of free radicals play a crucial role in the photocatalytic activity of ZnO/CdS heterostructures, and their concentration should be carefully controlled to achieve optimal performance.

## 5. Current State of Research on ZnO/CdS Heterostructures

Current research on ZnO/CdS heterostructures for photocatalysis has been actively pursued and has shown promising results. To the best of my knowledge, there are some key strategies which influence the photocatalytic performance of ZnO/CdS heterostructures including the synthesis method, morphology control, bandgap tuning, and interface engineering. Various synthesis methods have been explored to fabricate ZnO/CdS heterostructures with controlled morphologies and compositions. These methods include chemical precipitation, microwave, hydrothermal, solvothermal, and SILAR method. Each method offers advantages in terms of simplicity, scalability, and control over heterostructure properties. Researchers have focused on tailoring the morphology of ZnO/CdS heterostructures to enhance their photocatalytic performance. Morphological variations, such as nanorods, nanowires, nanosheets, nanoparticles, and hierarchical structures, have been synthesized and characterized. These unique morphologies provide a high surface area, improved charge separation, and efficient light absorption, leading to enhanced photocatalytic activity. The band alignment and interface between ZnO and CdS significantly influence the charge separation and transfer processes. Researchers have investigated strategies to optimize the energy level alignment and interface engineering through heterojunction formation or surface modification. By tuning the band alignment, the photogenerated charge carriers can be effectively separated and transferred, reducing charge recombination, and improving photocatalytic efficiency.

Enhancing the visible-light-driven photocatalytic activity of ZnO/CdS heterostructures has been a major focus. Various approaches, such as elemental doping, co-doping, or sensitization with noble metal nanoparticles, have been explored to extend the light absorption range and improve the utilization of solar energy. These strategies enable efficient visible-light-driven photocatalytic reactions, expanding the range of potential applications. Extensive research has been conducted to understand the photocatalytic mechanisms and reaction pathways in ZnO/CdS heterostructures. Techniques such as transient absorption spectroscopy, time-resolved photoluminescence, and electrochemical analysis have been employed to investigate the charge carrier dynamics, surface reactions, and intermediates involved in the photocatalytic process. These studies contribute to a deeper understanding of the photocatalytic mechanisms and guide the design of more efficient heterostructures. ZnO/CdS heterostructures have been applied in various photocatalytic applications, including the degradation of organic pollutants, water splitting for hydrogen production, and CO2 reduction. Studies have demonstrated their high catalytic activity, selectivity, and stability in these applications. Additionally, efforts have been made to scale up the synthesis methods and optimize the photocatalytic systems for practical implementation. It is worth noting that research is an ongoing and dynamic field, and new developments continue to emerge beyond my knowledge cutoff. I encourage you to explore the recent scientific literature and academic journals to stay updated on the current state of research on ZnO/CdS heterostructures for photocatalysis.

## 6. Conclusions and Future Perspectives

This article takes an in-depth look at the various synthesis approaches of ZnO/CdS heterostructures as well as their use as an efficient catalyst to remove contaminants from wastewater and H_2_ generation. To enhance the performance of ZnO/CdS heterostructures, various methodologies of optimization such as morphological control, bandgap engineering, development of ternary heterostructures, and co-catalyst loading optimization are also clearly explained. From the available literature, the following conclusions can be drawn, and we also propose some future research areas. ZnO/CdS heterojunction fabricated by using the chemical precipitation method demonstrated improved photocatalytic activity in dye degradation as well as good hydrogen production rate. The grain size of the heterostructures significantly enhances the H_2_ production. Therefore, in this method, to control the grain size, various ternary elements, such as Fe and GO, are doped into the ZnO/CdS heterostructures. The hydrogen production rate in ZnO/CdS heterostructures fabricated from the microwave method is higher than the other synthesis methods. The homogenous nucleation and quick crystallite growth and formation of a regulated size distribution of crystallite ZnO heterostructures in this method significantly enhanced the H_2_ evaluation. Compared to other conventional methods, heterostructures fabricated by the hydrothermal method have a high degree of crystallinity, homogeneous stoichiometry, and defect-free nanostructure. In this method, the size, morphology, and bandgap are significantly controlled by varying the experiment parameters such as the reaction time and temperature. A higher degree of crystallinity of ZnO/CdS heterostructures formed from the hydrothermal process offers the maximum efficiency for photocatalytic degradation. Furthermore, binary or ternary heterostructures with sizable surface areas and well-ordered morphologies are better constructed using the SILAR method.

However, it is impossible to say with certainty which synthesis technique is “best”. This evaluation offers insightful information that helps authors choose the best approach for their application. It is advised that writers take these things into account while creating nanocomposites in order to obtain a superior heterostructure interface for quick electron–hole charge transfer, which leads to enhanced photocatalytic activity. Researchers must concentrate on the choice of synthesis method in order to improve the photocatalytic performance of ZnO/CdS heterostructures. The formation of Z- and S-scheme photocatalysts by ZnO/CdS heterostructures can further boost their photocatalytic activity despite the lack of research in this field. While Z-scheme photocatalysts provide efficient charge separation by establishing an electron–hole transfer channel between two different semiconductors, S-scheme photocatalysts increase light absorption and utilization efficiency by employing a cascade of energy levels between two semiconductors. Therefore, the creation of Z- and S-scheme ZnO/CdS heterostructure photocatalysts is essential for improving photocatalytic activity and fostering the use of these catalysts in real-world energy conversion and environmental remediation applications. Furthermore, the creation of ternary heterostructures based on ZnO/CdS received less focus in earlier investigations. Therefore, it is suggested that researchers concentrate on this region. Overall, the suggested method will make it possible to create new and improved materials that may be used in a diverse field, such as energy and environmental sciences. The creation of robust and long-lasting photocatalysts can make it easier for them to be used practically in a variety of industries and contribute to the advancement of green and sustainable technology.

ZnO/CdS heterostructures have shown great potential for photocatalytic applications, and their prospects are promising. Ongoing research aims to further boost the photocatalytic performance of ZnO/CdS heterostructures by optimizing their morphology, composition, and surface properties. This includes developing novel nanostructured architectures, such as core–shell, heterojunctions, or hybrid composites, to improve light absorption, charge separation, and reaction kinetics. By tailoring the heterostructure design, it is possible to achieve a superior photocatalytic performance for various applications. Future research will focus on fine-tuning the band alignment and energy levels of the heterostructure components to enable efficient visible-light-driven photocatalysis. ZnO/CdS heterostructures hold significant potential for environmental remediation applications. They can be employed for the decolorization of organic pollutants, namely dyes, pesticides, and pharmaceutical compounds, from water and wastewater. Future studies will explore the design of ZnO/CdS heterostructures with improved catalytic activity, selectivity, and stability for efficient pollutant removal. Additionally, efforts will be made to scale up the production and optimize the photocatalytic systems for real-world applications. ZnO/CdS heterostructures can also be utilized in energy conversion and storage devices. For instance, they can serve as photoanodes in photoelectrochemical cells for water splitting to produce hydrogen, a clean and renewable fuel. Future research will focus on enhancing the efficiency and stability of ZnO/CdS-based photoelectrodes through morphology control, surface engineering, and catalyst integration. Furthermore, ZnO/CdS heterostructures can be explored for applications in solar cells, batteries, and supercapacitors to improve their energy conversion and storage capabilities. In summary, the prospects of ZnO/CdS heterostructures for photocatalytic applications involve further improving their photocatalytic activity, extending their spectral response, advancing environmental remediation and energy conversion technologies, exploring emerging applications, and optimizing their performance for real-world implementation. Continued research and development efforts in this field are expected to unlock the full potential of ZnO/CdS heterostructures in addressing energy and environmental challenges.

## Figures and Tables

**Table 2:** Advantages and disadvantages of different synthesis methods for ZnO/CdS heterostructures.

Synthesis Method	Advantages	Disadvantages
Coprecipitation	High yield, low cost, good control over composition, uniform particle size distribution, enhanced reactivity, easy scalability, and versatility.	Difficulty in controlling the particle size, sensitivity to reaction conditions, formation of secondary phase, and particles agglomeration.
Microwave	Fast reaction rate, high energy efficiency, scalability, high purity, and homogeneity.	Uneven heating, limited control over reaction conditions, defect formation, and risk of operation.
Hydrothermal	Controlled size and morphology, high crystallinity, low temperature synthesis, enhanced photocatalytic activity, and easy scalability.	Limited material options, a complex experimental setup, slow reaction rates, difficulty in controlling stoichiometry, and limited control over crystal orientation.
Solvothermal	Controlled size and morphology, high purity, enhanced photocatalytic activity, versatility, and easy scalability.	High temperature and pressure requirements, use of hazardous solvents, long synthesis time, difficulty in controlling stoichiometry, and high energy consumption.
SILAR	Low cost, good control over film thickness, large surface area, high purity, and room temperature synthesis.	Slow deposition rate, limited film thickness, poor control over composition, difficulty in achieving uniformity, and limited applicability to more complex nanocomposites.

**Table 5:** Advantages and disadvantages of different types of ZnO/CdS heterostructures.

Type of Heterostructure	Advantages	Disadvantages
p-n	Efficient charge separation, simple fabrication, and enhanced optoelectronic device performance.	Limited light absorption and band alignment challenges.
Type-I	Efficient charge separation, simple fabrication, and potential applications in the fields of photocatalysis, solar cells, and optoelectronic devices.	Limited light absorption and possibility of recombination of charge carriers.
Type-II	Broad spectrum light absorption, efficient charge separation, improved photocatalytic efficiency, optoelectronic performance, and solar cell efficiency.	Reduced carrier mobility, requiring precise control over band alignment and interface structures.
Type-III	Unique band alignment, enhanced charge separation, and potential for high-performance devices.	Limited light absorption, complex design, and challenging fabrication.
Z-Scheme	Efficient charge transfer, expanded light absorption, and versatile applications in photocatalysis, solar cells, and other energy conversion devices due to their efficient charge transfer and enhanced performance.	Complex design and fabrication, performance-dependence on mediator materials.
S-Scheme	Simple design compared to the Z-scheme, direct hole transfer, enhanced photocatalytic and photovoltaic characteristics	Limited charge transport and potential band alignment challenges.

## Data Availability

Not applicable.
